# Benefit of continuous kidney replacement therapy for managing tumor lysis syndrome in children with hematologic malignancies

**DOI:** 10.3389/fonc.2023.1234677

**Published:** 2023-08-18

**Authors:** Ashlea Anderson, Laurie Shoulders, Vinson James, Emily Ashcraft, Cheng Cheng, Raul Ribeiro, Lama Elbahlawan

**Affiliations:** ^1^ Division of Critical Care Medicine, St. Jude Children’s Research Hospital, Memphis, TN, United States; ^2^ Department of Nursing, Intensive Care Unit, St. Jude Children’s Research Hospital, Memphis, TN, United States; ^3^ Division of Pediatric Nephrology, Department of Pediatrics, LeBonheur Children’s Hospital, Memphis, TN, United States; ^4^ Department of Biostatistics, St. Jude Children’s Research Hospital, Memphis, TN, United States; ^5^ Department of Oncology, St. Jude Children’s Research Hospital, Memphis, TN, United States

**Keywords:** TLS, tumor lysis, CKRT, AKI, dialysis, pediatrics, hematologic malignancy

## Abstract

**Introduction:**

Tumor lysis syndrome (TLS) is often diagnosed in children with hematological malignancies and can be life threatening due to metabolic disturbances. Continuous renal replacement therapy (CKRT) can reverse these disturbances relatively quickly when conventional medical management fails. Our objective was to investigate the benefit of CKRT in the management of TLS in children admitted to the intensive care unit with hematologic malignancies. In addition, we sought to assess risk factors for acute kidney injury (AKI) in the setting of TLS.

**Methods:**

Retrospective review of all children admitted to the intensive care unit with TLS who received CKRT from January 2012 to August 2022.

**Results:**

Among 222 children hospitalized with TLS from January 2012 to August 2022, 20 (9%) underwent CKRT to manage TLS in the intensive care unit. The patients’ median age was 13 years (range 3-17 y), and most were males (18/20). T-cell acute lymphoblastic leukemia was the most common diagnosis (n=10), followed by acute myeloid leukemia (n=4), Burkitt lymphoma (n=4), and B-cell acute lymphoblastic leukemia (n=2). Five patients required mechanical ventilation, and 2 required vasopressors. The most common indication for CKRT was hyperphosphatemia, followed by, hyperuricemia, and hyperkalemia. All metabolic abnormalities corrected within 12 h of initiation of CKRT. CKRT courses were brief, with a median duration of 2 days (range 1-7 days). Having higher serum phosphorus levels 12 h preceding CKRT was significantly associated with severe acute kidney injury (AKI). The median phosphorus level was 6.4 mg/dL in children with no/mild AKI and 10.5 mg/dL in children with severe AKI (p=0.0375). Serum uric acid levels before CKRT were not associated with AKI. All children survived to hospital discharge, and the one-year survival rate was 90%.

**Conclusion:**

CKRT is safe in children with hematologic malignancies with severe TLS and reverses metabolic derangements within 6-12 h. Most patients had AKI at the initiation of CKRT but did not require long-term kidney replacement therapy. Hyperphosphatemia before initiation of CKRT is associated with higher risk of AKI.

## 1 Introduction

Tumor lysis syndrome (TLS) is a serious and life-threatening condition that is associated with hematologic malignancies. TLS occurs due to rapid breakdown of malignant cells either spontaneously or after the initiation of chemotherapy. This rapid breakdown leads to the release of intracellular potassium, phosphate, nucleic acids at a high rate that overwhelm the normal homeostatic mechanism for removing these byproducts. As a result, children will manifest laboratory TLS characterized by hyperuricemia, hyperkalemia, hyperphosphatemia, and hypocalcemia. The ensuing metabolic derangements can result in serious complications (Clinical TLS) such as acute kidney injury (AKI), arrhythmias, and seizures (1). AKI is typically induced by the deposition of uric acid or xanthine crystals in the renal tubules or by calcium-phosphate crystals deposition due to hyperphosphatemia. Therefore, prevention and prompt management of TLS is warranted, especially in patients at high risk of TLS. Medical management includes aggressive hydration at 1.5-2 times normal maintenance rate, with close monitoring of serum levels of potassium, phosphorus, calcium, and uric acid. In addition, rasburicase, a recombinant urate oxidase that converts uric acid to allantoin which is 10 times more soluble in water than uric acid, is prescribed to children with hyperuricemia. Management and outcome data about TLS in children is scarce (2, 3). Continuous kidney replacement therapy (CKRT) is utilized in severe cases of TLS to remove these solutes. CKRT provides slow and continuous removal of solutes which is more physiologic than intermittent hemodialysis and has less risk of rebound hyperphosphatemia and hyperkalemia.

Our objective was to investigate the benefit of CKRT in the management of TLS in children admitted to the intensive care unit (ICU) with hematologic malignancies. In addition, we sought to assess risk factors for AKI in the setting of TLS.

## 2 Methods

All children with hematologic malignancies admitted to St. Jude Children’s Research Hospital, a specialized pediatric hematologic-oncology hospital, from January 2012 to August 2022 were screened for TLS. Patients were included in the study if CKRT was initiated to manage TLS. This study was approved by our institutional review board. Laboratory TLS and clinical TLS were defined based on daily recorded laboratory and clinical values by using Cairo-Bishop criteria, (**Supplemental Figure 1**) (4). Clinical TLS was diagnosed in patients who had Laboratory TLS and one of these clinical findings: AKI, cardiac arrhythmias, or symptomatic hypocalcemia. AKI was defined and staged according to Kidney Disease Improving Global Outcomes (KDIGO) guidelines (**Supplemental Table 1**) (5). Severe AKI was defined as serum creatinine ≥ 2 times baseline (grade 2 and 3 per KDIGO guidelines).

The PrismaFlex CRRT system (Gambro/Baxter) was used with the continuous veno-venous hemodiafiltration (CVVHDF) treatment modality for all patients. All patients received continuous regional citrate infusion for anticoagulation and continuous systemic calcium infusion. Post-filter ionized calcium (Ica) levels were monitored every 2-4 hours.

### 2.1 Data collection

Daily collected laboratory test data included white blood cell count (WBC) and lactic dehydrogenase (LDH). In addition, the following laboratory values were measured at 6-hour intervals from 24 hours prior to 48 hours after CKRT initiation: uric acid, potassium, phosphorus, calcium, bicarbonate, blood urea nitrogen, and creatinine. Baseline serum creatinine level, when unknown, was imputed by the bedside Schwartz formula with an estimated glomerular filtration rate of 120 mL/min/1.73 m^2^ and the patient’s height (2).

### 2.2 Statistical analysis

Descriptive statistics are expressed in percentage for categorical variables and median (range) for continuous variables. The exact Wilcoxon rank-sum test was used to compare distributions of lab values by AKI status. The Wilcoxon signed-rank test was used to compare the differences between matched lab values before and post-CKRT. Spearman’s correlation was used to test the relationship between WBC and LDH at 1 and 2 days pre-CKRT and laboratory values pre-CKRT. Median (range) are reported for all statistical tests, and distribution-free 95% confidence intervals of the median difference between laboratory values are presented for comparisons of matched observations at different time points, as described by Hahn and Meeker (1991).

## 3 Results

Between January 2012 and August 2022, 222 children with hematologic malignancies were hospitalized with TLS. Of those, 20 (9%) required CKRT for management of their TLS in the ICU. **Table 1** summarizes the clinical characteristics of our cohort. The median age was 13 years (range, 3-17 y), and interestingly, most were male (18/20). The hematologic malignancy was T-cell acute lymphoblastic leukemia (ALL) in 10 children, acute myeloid leukemia (AML) or Burkitt lymphoma in 4 children each, and B-cell ALL in 2 children. A mediastinal mass was present in 8 children.

**Table 1:** Characteristics of patients with TLS and CKRT.

Clinical/Laboratory features	N (%) or Median (Min-Max)
Age (y)	13 (3–17)
Weight (Kg)	53.7 (17.3-130)
Sex	
Female	2 (10)
Male	18 (90)
Race	
African American	4 (21)
Caucasian	15 (79)
Primary diagnosis	
T-ALL	10 (50)
AML	4 (20)
Burkitt lymphoma	4 (20)
B-ALL	2 (10)
Chemical derangement	
Hyperphosphatemia	19 (95)
Hypocalcemia	18 (90)
Hyperuricemia	7 (35)
Hyperkalemia	2 (10)
Lab values 6 hours before CKRT	
BUN (mg/dL)	35.00 (17.0-81.0)
Cr (mg/dL)	1.28 (0.6-5.9)
Potassium (mmole/L)	4.60 (3.6-7.0)
Calcium (mg/dL)	6.70 (4.9-9.7)
Phosphorus (mg/dL)	9.70 (3.8-14.9)
Uric acid (mg/dL)	3.80 (0.2-29.7)
Peak Lab values	
Potassium (mmole/L)	5.5 (4-7.2)
Phosphorus (mg/dL)	10.7 (6.2-14.9)
Uric acid (mg/dL)	6 (2-29.7)
WBC† (10^3^/mm^3^)	41.60 (1.2-470.4)
LDH† (U/L)	2790 (439-10065)
Treatment prior to CKRT	
Fluid rate/BSA (mL/m^2^)	2597 (1193-3703)
Rasburicase	19 (95)
Allopurinol	9 (45)
Phosphate binders	17 (85)
Mechanical ventilation	5 (25)
Vasopressor support	2 (10)
Duration of ICU stay (days)	6.5 (3-36)
Duration of hospital stay (days)	12 (7-42)
Time from ICU admission to CKRT (days)	1.5 (0-7)
Duration of CKRT (days)	2 (1-7)

TLS, tumor lysis syndrome; CKRT, continuous kidney replacement therapy; ALL, acute lymphoblastic leukemia; AML, acute myeloblastic leukemia; BUN, blood urea nitrogen; Cr, creatinine; WBC, white blood cell count; LDH, lactate dehydrogenase; BSA, base surface area; ICU, intensive care unit.

† level one day prior to CKRT.

Of the 20 patients who underwent CKRT for TLS, 20 had laboratory TLS, and 18 had clinical TLS. At the time of initiation of CKRT, the most common chemical derangement was hyperphosphatemia in 95%, hypocalcemia in 90%, hyperuricemia in 35%, and hyperkalemia in 10%. Median serum phosphorus level was 9.7 mg/dL (range, 3.8-14.9 mg/dL) before start of CKRT (**Table 1**). LDH levels were elevated, with a median level of 2790 U/L (range, 439-10065 U/L).

TLS management included hydration with a median fluid volume of 2597 mL per m^2^ daily. In addition, rasburicase was administered to 19 of the 20 patients (95%), allopurinol to 9 (45%), and phosphate binders to 17 (85%).

### 3.1 CKRT course

**Figure 1:**
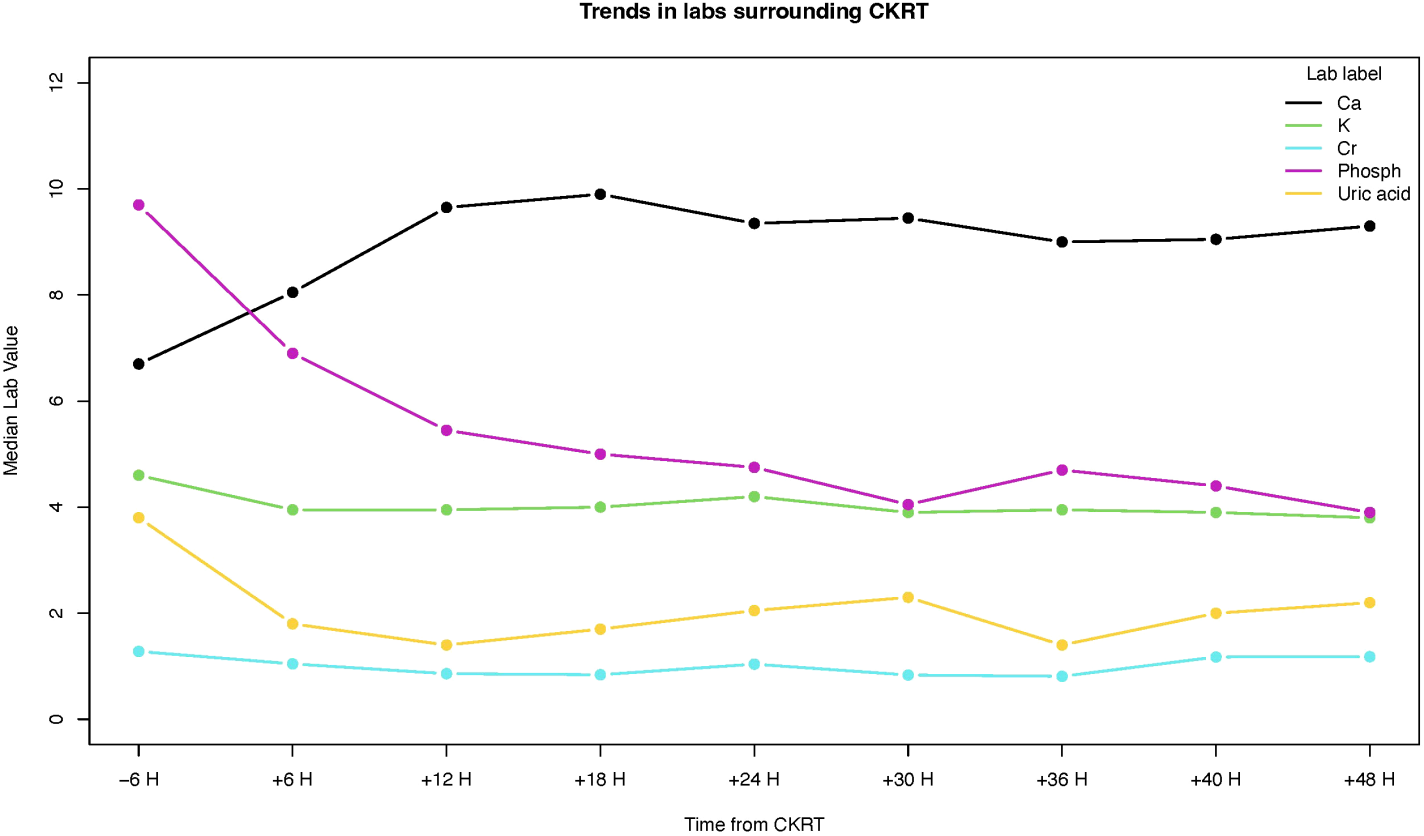
Changes in serum levels of calcium (Ca), potassium (K), creatinine (Cr), phosphorus (phosph), and uric acid.

In our cohort, 2 children had intermittent hemodialysis (IHD) before CKRT. Following the course of CKRT, 2 patients had one session of IHD, and one patient received IHD for 15 days. The median duration of CKRT course in our cohort was 2 d (range 1-7 d). Potassium, phosphorus, and uric acid levels dropped significantly within 6 h after starting CKRT (**Table 2**). Compared to the level 6 h before initiation, the median phosphorus level declined 2.7 mg/dL (p-value <0.0001) in 6 h and 5.05 mg/dL (p-value <0.0001) in 18 h post CKRT. All serum levels of potassium, phosphorus, uric acid levels normalized within 12 hours of CKRT initiation (**Figure 1**). None of the patients had CKRT-related complications.

**Table 2:** Median difference in serum levels 6 h before and up to 24 h post CKRT.

Serum level	Median Difference (95% CI)	p-value
K (mmole/L)		
6 h pre vs. 6 h post CKRT	0.70 (0.5, 0.9)	0.0040
6 h pre vs. 12 h post CKRT	0.80 (0.5, 1.3)	0.0010
6 h pre vs. 18 h post CKRT	0.65 (0.3, 1.1)	0.0143
6 h pre vs. 24 h post CKRT	0.80 (-0.3, 1.2)	0.1533
Phosphorus (mg/dL)		
6 h pre vs. 6 h post CKRT	2.70 (1.9, 3.7)	<.0001
6 h pre vs. 12 h post CKRT	4.80 (2.8, 5.7)	0.0001
6 h pre vs. 18 h post CKRT	5.05 (3.1, 6.2)	<.0001
6 h pre vs. 24 h post CKRT	4.80 (1.5, 7.6)	0.0015
Uric acid (mg/dL)		
6 h pre vs. 6 h post CKRT	1.80 (0.1, 7.7)	0.0090
6 h pre vs. 12 h post CKRT	3.10 (-0.2, 8.8)	0.0023
6 h pre vs. 18 h post CKRT	3.40 (0.6, 8.8)	0.0010
6 h pre vs. 24 h post CKRT	3.00 (-0.2, 10.2)	0.0210
Calcium (mg/dL)		
6 h pre vs. 6 h post CKRT	0.0057
6 h pre vs. 12 h post CKRT	-2.60 (-3.6,-1.1)	<.0001
6 h pre vs. 18 h post CKRT	-3.35 (-3.8, -1.9)	<.0001
6 h pre vs. 24 h post CKRT	-3.00 (-3.8, -1.4)	0.0012
BUN (mg/dL)		
6 h pre vs. 6 h post CKRT	4.00 (-4.0, 13.0)	0.0927
6 h pre vs. 12 h post CKRT	16.00 (7.0, 23.0)	0.0010
6 h pre vs. 18 h post CKRT	17.00 (6.0, 27.0)	0.0004
6 h pre vs. 24 h post CKRT	12.00 (1.0, 30.0)	0.0039
Cr (mg/dL)		
6 h pre vs. 6 h post CKRT	0.25 (0.1, 0.4)	0.0002
6 h pre vs. 12 h post CKRT	0.48 (0.2, 0.8)	0.0017
6 h pre vs. 18 h post CKRT	0.68 (0.4,0.9)	0.0005
6 h pre vs. 24 h post CKRT	0.89 (0.4, 2.3)	0.0043

CKRT, continuous kidney replacement therapy; K, potassium; BUN, blood urea nitrogen; Cr, creatinine.

### 3.2 Risk factors for TLS

We investigated whether WBC or LDH levels one day before the start of CKRT were associated with hyperkalemia, hyperphosphatemia, duration of CKRT, or duration of ICU stay (**Supplemental Tables 2**, **3**). Higher LDH levels correlated with higher uric acid levels 12 h prior to CKRT (p-value 0.0803 | Rho = 0.45).

### 3.3 Risk factors for AKI

**Figure 2:**
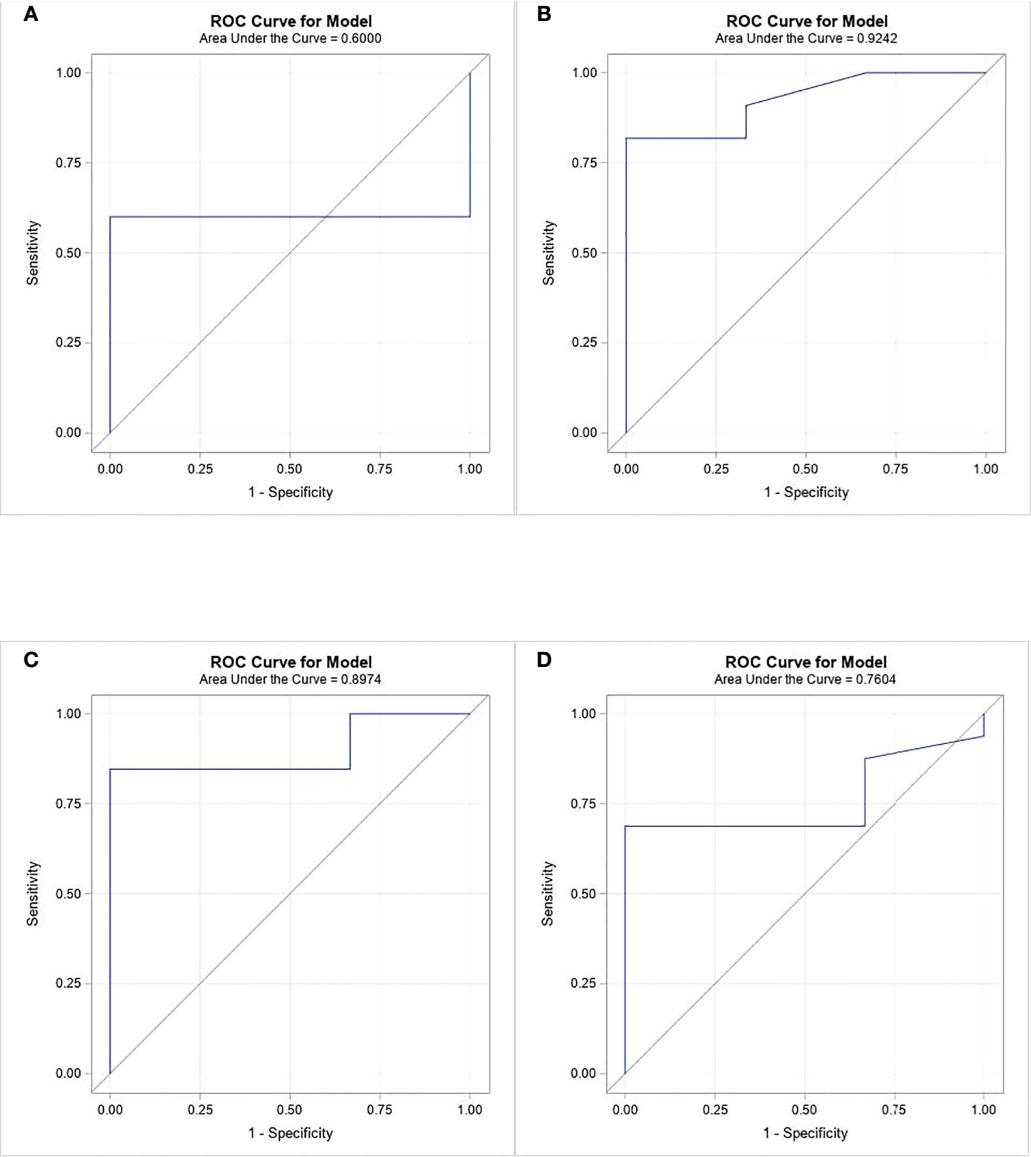
ROC analysis of phosphorous levels at 4 time periods prior to CKRT initiation as a predictor of severe AKI. **(A)** at 24 hours, **(B)** at 18 hours, **(C)** at 12 hours, **(D)** at 6 hours.

Severe AKI (grade 2 and 3) was present in 17 patients (85%). Risk factors for development of AKI were examined. Uric acid, phosphorus, and LDH serum levels in patients with no/mild AKI were compared to those of patients with severe AKI (**Table 3**). Serum uric acid levels 12 h and 6 h preceding CKRT were not associated with AKI. However, higher serum phosphorus levels 12 h preceding CKRT were significantly associated with severe AKI. The median phosphorus level was 6.4 mg/dL in children with no/mild AKI but 10.5 mg/dL in children with severe AKI (p-value 0.0375). Furthermore, ROC analysis revealed that phosphorus level at 18 h prior to CKRT was the best predictor of severe AKI (ROC = 0.9 SE = 0.075 95% CI = 0.77 – 1.07) (**Figure 2**). Children in whom severe AKI developed had similar durations of ICU or hospital stay as those in whom severe AKI did not develop.

**Table 3:** Comparison of patients with no/mild AKI and those with severe AKI.

Laboratory measure	Median (min, max)		P value
	No/Mild AKI (Stage 0,1) n=3	Severe AKI (Stage 2,3) n=17	
Uric acid (mg/dL)			
6 h pre CKRT	3.70 (2.3, 9.0)	3.90 (0.2, 29.7)	0.723
12 h pre CKRT	2.40 (1.1, 7.7)	5.00 (0.9, 17.7)	0.3643
Phosphorus (mg/dL)			
6 h pre CKRT	8.20 (7.3, 8.9)	10.65 (3.8, 14.9)	0.1785
12 h pre CKRT	6.40 (5.1,7.1)	10.50 (5.8, 12.4)	0.0375
Calcium (mg/dL)			
6 h pre CKRT	7.30 (6.6, 7.5)	6.65 (4.9, 9.7)	0.3199
12 h pre CKRT	7.40 (7.3, 7.5)	7.10 (5.6, 9.0)	0.2964
LDH 1 d pre CKRT (U/L)	2303.00 (2147.0, 2790.0)	2340.50 (351.0, 10065.0)	0.9577
Duration of hospital stay (d)	16.00 (9.0, 21.0)	12.00 (7.0, 42.0)	0.4825
Duration of ICU stay (d)	7.00 (4.0, 8.0)	6.00 (3.0, 36.0)	0.8491

AKI, acute kidney injury; CKRT, continuous kidney replacement therapy; LDH, lactate dehydrogenase; ICU, intensive care unit.

### 3.4 ICU course

Five children (25%) received invasive mechanical ventilation for acute respiratory failure, with a median duration of 4 days. Two patients (10%) were on vasopressor support. The median duration of ICU stay was 6.5 d, whereas the median duration of hospital stay was 12 d. All children survived to ICU and hospital discharge. The overall survival rate at one year was 90%. Renal function improved in all patients, and none required long-term dialysis.

## 4 Discussion

This study analyzed CKRT courses and outcome in a cohort of 20 children admitted to the ICU with hematologic malignancies and severe TLS. Of all 222 children hospitalized with hematologic malignancies and TLS, only 9% required CKRT. CKRT was successful in abating the metabolic derangements within 6 hours of initiation, and serum levels of potassium, phosphorus, and uric acid declined to normal levels within 12 h of the CKRT course. There is limited data on the use of CKRT for TLS especially for pediatric patients. In an adult cohort of 153 patients with newly diagnosed hematologic malignancies who were at high risk for TLS, 30.7% developed TLS. Of those in whom TLS developed, 27 required kidney replacement therapy (KRT) (17.6%), and 17 required CKRT (11%) (6). In another cohort of adults with TLS admitted to ICU, KRT was utilized in 54.2% of patients, and the incidence of AKI was 80.4% (7). In our cohort, the most common indication for CKRT was hyperphosphatemia, and only one-third had hyperuricemia at the initiation of CKRT. This is not surprising in the rasburicase era, as most patients receive rasburicase early during a TLS course to prevent renal damage induced by uric acid crystal deposition in the renal tubules. Indeed, 95% of our cohort received rasburicase before CKRT. A prospective pediatric study of 76 patients with B-cell non-Hodgkin lymphoma found rasburicase to be effective at normalizing 86% and 100% of the uric acid levels in patients at 24 and 72 h respectively (8). In addition, Darmon et al. reported serum phosphorus level to be the main risk factor for clinical TLS, with a 5-fold increase in risk of clinical TLS with each1 mmole increase in phosphorus level (6).

In our cohort of severe TLS requiring CKRT, most patients were males (90%). This observation was reported in previous TLS cohorts: in a cohort of 8 children who required renal replacement therapy due to TLS, 87.5% were male (9). In addition, in a cohort of 153 adults with cancer and TLS admitted to the ICU, 69% were male, and AKI occurred at a rate of 86% in male patients. Being male is associated with higher risk of AKI (OR=6.79, IC 95% 2.59-19.44) (7, 9). Prospective large cohorts are needed to examine and confirm the association of male sex with severe TLS and AKI. If confirmed, then being male should be considered a risk factor for severe clinical TLS. Of note, male sex has been reported in previous studies to confer a higher risk of AKI requiring dialysis (10, 11). In a large cohort of hospitalized patients with AKI, AKI-D was 2.19 times more likely to develop in men than in women (11).

The reported prevalence of AKI in the setting of TLS is high and ranges from 64-80% (6, 7). Severe AKI was observed in 85% of our cohort. Our findings indicate that hyperphosphatemia contributes to AKI in this pediatric cohort. Children with AKI had significantly higher phosphorus levels before initiation of CKRT (6.4 mg/dL in children with no/mild AKI vs.10.5 mg/in those with severe AKI, p-value 0.0375). These findings are similar to those previously described in adult cohorts. In a large cohort of 120 adults with hematologic malignancies and TLS, AKI developed in 56, and phosphate was strongly associated with AKI (Hazard ratio of 1.76 per 0.5 mmole/L increase in phosphate) (12). As in our cohort, uric acid levels did not contribute to AKI. On the basis of these findings, we suggest that rapid rise in phosphorus levels should alert clinicians to consider the initiation of CKRT in these situations to prevent developing AKI or progression of an existing AKI. Abdel-Nabey et al. reported the practice of KRT initiation in patients with TLS admitted to the ICU with a phosphorus level of > 7.7 mg/dL or when the phosphorus level increase is >3 mg/dL every 6 h (7). This practice is reasonable considering the strong association of hyperphosphatemia with AKI in this pediatric cohort and in adult cohorts (6, 12).

The reported overall mortality rate of patients with TLS ranges from 15 to 35% (6, 13, 14). In our cohort, all patients survived to ICU and hospital discharge even though 85% had severe AKI and 25% had acute respiratory failure. This outcome suggests that early intervention and CKRT provide benefit and improve outcome in this population. In addition, in our cohort, the overall mortality at 1 year was low (10%).

The limitations of our study include its retrospective design, small population, and absence of a control group. However, our study describes the largest cohort of children with TLS and hematologic malignancies who were treated by CKRT. Prospective randomized, controlled studies are needed to outline the benefit of early KRT initiation to prevent AKI in this high-risk population.

## 5 Conclusion

CKRT is safe in children with hematologic malignancies with severe TLS and reverses metabolic derangements within 6-12 h. Most patients had AKI at the time of initiation of CKRT but did not require long-term KRT. Hyperphosphatemia before initiation of CKRT was associated with AKI; thus, rapidly rising phosphorus level can indicate the need for CKRT. Male sex seems to be associated with a higher risk of TLS requiring dialysis. The results of prospective multicenter studies may identify a cut-off phosphorus value at which to start CKRT.

## Data availability statement

The raw data supporting the conclusions of this article will be made available by the authors, without undue reservation.

## Ethics statement

The studies involving human participants were reviewed and approved by IRB at St. Jude Children’s Research Hospital. Written informed consent for participation was not provided by the participants’ legal guardians/next of kin because: Retrospective data collection.

## Author contributions

LE contributed to planning, writing, and editing the manuscript. AA and LS contributed to data collection, writing, and editing the manuscript. VJ contributed to data collection and editing the manuscript, CC and EA contributed to data analysis and manuscript writing, RR contributed to planning and editing the manuscript. All authors contributed to the article and approved the submitted version.

## Supplementary material

Supplementary Figure 1 Definition of laboratory and clinical TLS.
