# Supplementary material for: Benefit of continuous kidney replacement therapy for managing tumor lysis syndrome in children with hematologic malignancies
Source: Front Oncol. 2023 Aug 18;13:1234677. doi: 10.3389/fonc.2023.1234677 (PMC10471890; doi:10.3389/fonc.2023.1234677)
Supplement: Supplementary Figure 1 — Definition of laboratory and clinical TLS. [file Table_1.docx]

| **KDIGO stage** |  | **KDIGO staging** |
| --- | --- | --- |
| **Stage 1** | **Scr** | 1.5–1.9x Bl OR ≥0.3 mg/dL |
|  | **UO** | <0.5 mL/kg/h for 6-12 h |
| **Stage 2** | **Scr** | 2.0–2.9x Bl |
|  | **UO** | <0.5 mL/kg/h for 12-24 h |
| **Stage 3** | **Scr** | 3.0x Bl OR ≥4.0 mg/dL  OR KRT  OR eGFR <35 mL/min/1.73 m^2^ |
|  | **UO** | <0.3 mL/kg/h for ≥24 h  OR Anuria for ≥12 h |
|  |  |  |

**Table 1. KDIGO staging criteria for AKI**

KDIGO, Kidney Disease Improving Global Outcomes; AKI, acute kidney injury; Scr, serum creatinine; Bl, baseline; UO, urine output; eGFR, estimated glomerular filtration rate; KRT, kidney replacement therapy
