# Supplementary material for: Benefit of continuous kidney replacement therapy for managing tumor lysis syndrome in children with hematologic malignancies
Source: Front Oncol. 2023 Aug 18;13:1234677. doi: 10.3389/fonc.2023.1234677 (PMC10471890; doi:10.3389/fonc.2023.1234677)
Supplement: Supplementary file 2 [file DataSheet_1.docx]

Figure 1. TLS definition in children. For diagnosis of Laboratory TLS: 2 or more elevated serum levels within 3 days before and 7 days after initiation of chemotherapy. For diagnosis of clinical TLS: Laboratory TLS and one of the criteria.
