# Supplementary material for: Benefit of continuous kidney replacement therapy for managing tumor lysis syndrome in children with hematologic malignancies
Source: Front Oncol. 2023 Aug 18;13:1234677. doi: 10.3389/fonc.2023.1234677 (PMC10471890; doi:10.3389/fonc.2023.1234677)
Supplement: Supplementary file 3 [file Table_2.docx]

| Variable |  | Median (Min,Max) | Rho | p-value |
| --- | --- | --- | --- | --- |
| LDH 1 d pre CKRT |  | 2311(351.0,10065.0) | 1 |  |
| K 6 h pre CKRT |  | 4.60 (3.6,7.0) | -0.11851 | 0.6395 |
| K 12 h pre CKRT |  | 5.00 (3.7,7.2) | -0.07069 | 0.7947 |
| Phosphorous 6 h pre CKRT |  | 10.10 (3.8,14.9) | 0.17872 | 0.4780 |
| Phosphorous 12 h pre CKRT |  | 9.70 (5.1,12.4) | 0.11479 | 0.6721 |
| Uric acid 6 h pre CKRT |  | 3.80 (0.2,29.7) | 0.18210 | 0.4696 |
| Uric acid 12 h pre CKRT |  | 4.80 (0.9,17.7) | 0.45000 | 0.0803 |
| Duration of hospital stay (d) |  | 12.00 (7.0,41.0) | -0.07543 | 0.7589 |
| Duration of ICU stay (d) |  | 6.00 (3.0,24.0) | -0.31676 | 0.1864 |
| Duration of CKRT (h) |  | 36.00 (13.0,97.3) | -0.16842 | 0.4907 |

Table 2 Spearman correlation between LDH 1 d pre-CKRT vs labs and clinical course

LDH, lactate dehydrogenase; CKRT, continuous kidney replacement therapy, K, potassium, ICU, intensive care unit
