# Supplementary material for: Benefit of continuous kidney replacement therapy for managing tumor lysis syndrome in children with hematologic malignancies
Source: Front Oncol. 2023 Aug 18;13:1234677. doi: 10.3389/fonc.2023.1234677 (PMC10471890; doi:10.3389/fonc.2023.1234677)
Supplement: Supplementary file 4 [file Table_3.docx]

Table 3. Spearman correlation between WBC 1-day pre CKRT vs labs and clinical course

WBC, white blood count; CKRT, continuous kidney replacement therapy, K, potassium, ICU, intensive care unit

| Variable |  | Median (Min,Max) | Rho | p-value |
| --- | --- | --- | --- | --- |
| WBC 1 d pre CKRT |  | 41.60(1.2,470.4) | 1 |  |
| K 6 h pre CKRT |  | 4.60(3.6,7.0) | -0.02718 | 0.9175 |
| K 12 h pre CKRT |  | 5.10(3.7,7.2) | 0.35063 | 0.2001 |
| Phosphorous 6 h pre CKRT |  | 9.70(3.8,14.9) | -0.16800 | 0.5192 |
| Phosphorous 12 h pre CKRT |  | 8.90(5.1,12.4) | -0.05357 | 0.8496 |
| Uric acid 6 h pre CKRT |  | 3.80(0.2,29.7) | 0.11209 | 0.6794 |
| Uric acid 12 h pre CKRT |  | 5.00(0.9,17.7) | 0.16786 | 0.5499 |
| Duration of hospital stay (d) |  | 12.00(7.0,42.0) | -0.11606 | 0.6465 |
| Duration of ICU stay (d) |  | 6.50(3.0,36.0) | -0.10510 | 0.6781 |
| Duration of CKRT (h) |  | 33.00(13.0,97.3) | 0.01961 | 0.9384 |
